# The benefits of an integrated social medical insurance for health services utilization in rural China: evidence from the China health and retirement longitudinal study

**DOI:** 10.1186/s12939-021-01457-8

**Published:** 2021-05-24

**Authors:** Xiaojing Fan, Min Su, Yafei Si, Yaxin Zhao, Zhongliang Zhou

**Affiliations:** 1 School of Public Policy and Administration, Xi’an Jiaotong University, Xi’an, China; 2 School of Public Administration, Inner Mongolia University, Hohhot, China; 3 School of Risk & Actuarial Studies and CEPAR, University of New South Wales, Kensington, China; 4 School of Public Health, Xi’an Jiaotong University Health Science Centre, Xi’an, China

**Keywords:** Social medical insurance, Health services utilization, Equity, Rural China

## Abstract

**Background:**

Improving health equity is a fundamental goal for establishing social health insurance. This article evaluated the benefits of the Integration of Social Medical Insurance (ISMI) policy for health services utilization in rural China.

**Methods:**

Using the China Health and Retirement Longitudinal study (2011‒2018), we estimated the changes in rates and equity in health services utilization by a generalized linear mixed model, concentration curves, concentration indices, and a horizontal inequity index before and after the introduction of the ISMI policy.

**Results:**

For the changes in rates, the generalized linear mixed model showed that the rate of inpatient health services utilization (IHSU) nearly doubled after the introduction of the ISMI policy (8.78 % vs. 16.58 %), while the rate of outpatient health services utilization (OHSU) decreased (20.25 % vs. 16.35 %) after the implementation of the policy. For the changes in inequity, the concentration index of OHSU decreased significantly from − 0.0636 (95 % CL: −0.0846, − 0.0430) before the policy to − 0.0457 (95 % CL: −0.0684, − 0.0229) after it. In addition, the horizontal inequity index decreased from − 0.0284 before the implementation of the policy to − 0.0171 after it, indicating that the inequity of OHSU was further reduced. The concentration index of IHSU increased significantly from − 0.0532 (95 % CL: −0.0868, − 0.0196) before the policy was implemented to − 0.1105 (95 % CL: −0.1333, − 0.0876) afterwards; the horizontal inequity index of IHSU increased from − 0.0066 before policy implementation to − 0.0595 afterwards, indicating that more low-income participants utilized inpatient services after the policy came into effect.

**Conclusions:**

The ISMI policy had a positive effect on improving the rate of IHSU but not on the rate of OHSU. This is in line with this policy’s original intention of focusing on inpatient service rather than outpatients to achieve its principal goal of preventing catastrophic health expenditure. The ISMI policy had a positive effect on reducing the inequity in OHSU but a negative effect on the decrease in inequity in IHSU. Further research is needed to verify this change. This research on the effects of integration policy implementation may be useful to policy makers and has important policy implications for other developing countries facing similar challenges on the road to universal health coverage.

**Supplementary Information:**

The online version contains supplementary material available at 10.1186/s12939-021-01457-8.

## Background

Rural population health is attracting considerable attention from scholars all over the world and policy-makers in the mission to improve their health [1, 2]. Currently, under the global trend of aging, the healthcare issues arising from a rural aging population are among the major challenges to the soundness and development of China’s health system in the long term. Studies have found that the middle-aged and elderly have an altered functional capacity [3] and are a prevalence group for general illnesses, chronic non-communicable diseases, mental health disorders, and cancer [4, 5]; they will be the target group of health service utilization in the future [6]. It is important to pay attention to their health service utilization status and related improving strategies in order to prospectively respond to the issues of healthcare resource allocation due to the aging of the rural population.

To maintain the living standard of aging people and reduce their risk of poverty and ill health in later life, establishing a universal social medical insurance (SMI) has become an important strategy to improve this population’s utilization of healthcare services [7, 8]. SMI can improve this population’s health by reducing out-of-pocket costs and increasing people’s health services utilization [7, 9, 10]. The Chinese government has made many efforts over the past few decades to improve SMI for the rural population. In 2003, China established the New Rural Cooperative Medical Scheme (NCMS) policy [11]. However, with rapid economic and social development, the NCMS policy has shown a large gap between the level of insurance for urban residents and inconvenient reimbursement in urban and rural areas [8, 12–14]. In order to solve these problems and advance the universal SMI, some provinces started to improve rural medical insurance by integrating rural and urban residents’ medical insurance on a trial basis from 2010 onward. In 2016, the State Council of China issued the “Opinion on the Integration of Basic Medical Insurance Systems between Urban and Rural Residents” report on January 12, proposing to integrate the NCMS and Urban Residents’ Basic Medical Insurance (URBMI) into the same medical insurance in other provinces based on the experience with the NCMS, URBMI, and trial provinces [15, 16]. Therefore, it is hypothesized that health services utilization in rural areas will increase after implementing the Integration of Social Medical Insurance (ISMI) policy.

Inequity exists between and within countries around the world in terms of healthcare access and utilization, quality of care, and health outcomes [17]. Improving health equity is a stated goal of many governments and international organizations, and it is a fundamental goal in establishing SMI. A large body of literature has explored the impact of socioeconomic characteristics, such as past economic status and education on health service inequalities [18–20]. Groups with better socioeconomic characteristics tend to have better access to health services and are able to afford higher-quality services when accessing health services compared to disadvantaged groups. This means that, without targeted policy implementation, inequities in health services will grow. To assist the government and policy makers in implementing effective scientific and policy interventions, this study conducts an effect analysis of the ISMI policy being implemented with the aim of providing scientific data to support further policy implementation.

Previous studies have mainly focused on the analysis of policy theory [16, 21]; some have demonstrated that ISMI policy increased inpatient care [22], the rate of medical return [23], and the number of outpatient visits in rural areas [24], while several scholars have described the experiences, willingness, satisfaction, and challenges of integrating in pilot areas [16, 21, 25]. However, it is not clear to date how the inequity in access to health services changed after the integration of SMI, especially among vulnerable groups. This study aims to explore the effect of ISMI policy on the change in equity in health services utilization in rural areas. The importance of the paper is twofold: firstly, it contributes to the limited evidence on inequity in health service utilization under ISMI, paying particular attention to vulnerable groups; secondly, the paper provides rich explanatory data on the basis of the China Health and Retirement Longitudinal study (CHARLS), which was a nationwide survey administered in representative regions of China before and after the implementation of the ISMI policy. Our findings will provide empirical support for future policy formulation on SMI integration in China and offer lessons to countries facing similar challenges.

## Methods

### Data source

We used data obtained from the 2011 and 2018 CHARLS before and after the introduction of China’s ISMI policy. The reason was as follows: as only some provinces had been implementing the ISMI policy since around 2010, the pre-policy data were selected from the 2011 CHARLS; as most provinces had been implementing the ISMI policy since the end of 2016, the post-policy data were selected from the 2018 CHARLS. The CHARLS was hosted by the National Development Research Institute of Peking University and was jointly implemented by the Chinese Social Science Research Center of Peking University and the University Youth League Committee [26]. The CHARLS utilized a multi-stage probability proportional scale sampling method to randomly select Chinese middle-aged and elderly people and their spouses from 150 counties and 450 communities (villages) across 28 provinces as respondents [27]. Questionnaires and medical examinations were conducted by face-to-face household surveys, which included information on demographics, physical examination, biochemical tests, socioeconomic status, health status and functioning, health care and insurance.

This study focused on rural Hukou residents aged 45 years or older. Only participants who had experienced the NCMS were selected as the sampling unit of interest in 2011. In 2018, participants who had experienced the ISMI were selected, although participants who had experienced NCMS were also selected based on the fact that some participants do not know the name of the ISMI. Finally, data from 12,145 participants in 2011 and 12,705 participants in 2018 were utilized for the analysis.

### Key indicators

In this study, two outcome variables were used to reflect health services utilization: the outpatient health services utilization (OHSU) and inpatient health services utilization (IHSU). In keeping with previous studies and the content of the CHARLS on health services utilization [28], OHSU, whose serial number in the questionnaire was “ED001”, was measured as the probability of receiving an outpatient visit in the last month, while IHSU was numbered “EE003” in the questionnaire and was measured as the probability of receiving an inpatient visit in the past year. They were two binary variables, where 0 denoted “no” and 1 denoted “yes”. The rate of OHSU and IHSU were calculated as follows:

$$\text{O}\text{H}\text{S}\text{U} \text{r}\text{a}\text{t}\text{e}=\frac{\text{n}\text{u}\text{m}\text{b}\text{e}\text{r} \ \text{o}\text{f} \ \text{p}\text{a}\text{r}\text{t}\text{i}\text{c}\text{i}\text{p}\text{a}\text{n}\text{t}\text{s} \ \text{r}\text{e}\text{c}\text{e}\text{i}\text{v}\text{i}\text{n}\text{g} \ \text{a}\text{n} \ \text{o}\text{u}\text{t}\text{p}\text{a}\text{t}\text{i}\text{e}\text{n}\text{t} \ \text{v}\text{i}\text{s}\text{i}\text{t} \ \text{i}\text{n} \ \text{t}\text{h}\text{e} \ \text{l}\text{a}\text{s}\text{t} \ \text{m}\text{o}\text{n}\text{t}\text{h}}{\text{n}\text{u}\text{m}\text{b}\text{e}\text{r} \ \text{o}\text{f} \ \text{p}\text{a}\text{r}\text{t}\text{i}\text{c}\text{i}\text{p}\text{a}\text{n}\text{t}\text{s} \ \text{s}\text{u}\text{r}\text{v}\text{e}\text{y}\text{e}\text{d}}\times 100\%$$

$$\text{I}\text{H}\text{S}\text{U} \text{r}\text{a}\text{t}\text{e}=\frac{\text{n}\text{u}\text{m}\text{b}\text{e}\text{r} \ \text{o}\text{f} \ \text{p}\text{a}\text{r}\text{t}\text{i}\text{c}\text{i}\text{p}\text{a}\text{n}\text{t}\text{s} \ \text{r}\text{e}\text{c}\text{e}\text{i}\text{v}\text{i}\text{n}\text{g} \ \text{a}\text{n} \ \text{i}\text{n}\text{p}\text{a}\text{t}\text{i}\text{e}\text{n}\text{t} \ \text{v}\text{i}\text{s}\text{i}\text{t} \ \text{i}\text{n} \ \text{t}\text{h}\text{e} \ \text{p}\text{a}\text{s}\text{t} \ \text{y}\text{e}\text{a}\text{r}}{\text{n}\text{u}\text{m}\text{b}\text{e}\text{r} \ \text{o}\text{f} \ \text{p}\text{a}\text{r}\text{t}\text{i}\text{c}\text{i}\text{p}\text{a}\text{n}\text{t}\text{s} \ \text{s}\text{u}\text{r}\text{v}\text{e}\text{y}\text{e}\text{d}}\times 100\%$$

The core independent variable was the ISMI policy. It was hypothesized that the rate of OHSU and IHSU in rural areas would increase after implementing the ISMI policy. The following variables were considered covariates, and all of which likely affected the association between policy and health services utilization: the rural participants’ sex, age, education, economic and living status, sleeping hours, smoking status, alcohol consumption, disability, and chronic pain and diseases.

### Analytic strategy

A generalized linear mixed model (GLMM) was employed to analyze the effect of the ISMI policy on health services utilization after controlling for a number of confounding factors based on previous studies but constrained by the variables collected in the CHARLS (Table 1) [19, 20, 29]. Odds ratios (ORs) with 95 % confidence limits (CLs) were derived from the GLMM. The policy variable was specified as the fixed effect, and the community where rural participants lived was a random effect.

**Table 1:** Basic characteristics (*n*=24,850)

Variables	Group	***N***	%
Policy	Before	12,145	48.87
	After	12,705	51.13
**Demographics**			
Sex	Male	11,612	46.75
	Female	13,228	53.25
Age (years)	45-50	4,329	17.42
	51-60	8,594	34.58
	61-70	7,510	30.22
	≥71	4,417	17.77
Education	Illiterate	7,606	30.62
	≤Elementary school	11,238	45.25
	≥Middle school	5,992	24.13
Economic status	Low	6,147	25.01
	Middle	12,289	50.01
	High	6,139	24.98
**Life style**			
Living status	Live with others	19,607	78.90
	Live alone	5,242	21.10
Sleeping hours	7-8 hours	8,932	35.94
	≤6 hours	12,499	50.30
	>8 hours	3,419	13.76
Smoking status	No	14,555	73.04
	Yes	5,373	26.96
Alcohol consumption	No	16,791	67.62
	Yes	8,042	32.38
**Health status**			
Disability	No	17,341	80.97
	Yes	4,075	19.03
Body pain	No	12,807	51.54
	Yes	12,043	48.46
Chronic diseases	No	11,134	44.80
	Yes	13,716	55.20

Concentration curves, concentration indices (CI), and their decomposition were applied to analyze the equity of OHSU and IHSU. Concentration curves and indices were used to measure the extent of economic status-related inequality in the distribution of OHSU and IHSU across the population [30, 31]. The inequality measured in this study using the CI was a relative inequality. A positive concentration index denoted that people with high economic status use more health services than their low-income counterparts do, whereas a negative index denoted the opposite. The CI formula was as follows:

$$C=\frac{2}{{\upmu }}COV\left(y, \gamma \right)$$,

where C is defined in terms of the covariance between the outcome variable (y) and the fractional ranks of household income (γ); µ is the mean of y.

Inequality can be further explained by decomposing the CI into its determining components; then, the horizontal inequity index (HI) can be computed by subtracting the contribution of needed variables (such as sex, age, and health status) from the concentration indexes of OHSU and IHSU [32]. The process was as follows: first, a regression model on the outcome variable (y) was established:

$${\text{y}}_{i}={a}^{m}+{\sum }_{k}{\beta }_{k}^{m}{x}_{ki}+{\mu }_{i}$$,

where ${\beta }_{k}^{m}$ is the marginal effect (dy/dx) of each x; ${\mu }_{i}$indicates the error term; the concentration index for y can be expressed as follows:

$$C={\sum }_{k}\left({\beta }_{k}\stackrel{-}{{x}_{k}}/\mu \right){c}_{k}+{GC}_{\epsilon }/\mu$$,

where ${\beta }_{k}$ is the marginal effect of ${x}_{k}$; $\stackrel{-}{{x}_{k}}$ and ${c}_{k}$ are the mean and the CI of ${x}_{k}$; µ is the mean of y; and ${GC}_{\epsilon }$ is the generalized CI for ε. This equation revealed the total CI, consistent with that of two components: the explained component and the residual component. The first component contained two elements: (1) elasticity ${\beta }_{k}\stackrel{-}{{x}_{k}}/\mu$ as a unit-free measure of association that indicates the amount of change in the dependent variable associated with a one-unit change in the explanatory variable; (2) ${c}_{k}$, the normalized CI of the K variable. ${GC}_{\epsilon }/\mu$ represents the unexplained component, which cannot be described by systematic variation in the determinants across economic groups. All data management and statistical analyses were performed on STATA statistical software version 14.0 (StataCorp LP, College Station, TX, USA).

## Results

### Change in rates

**Fig. 1:**
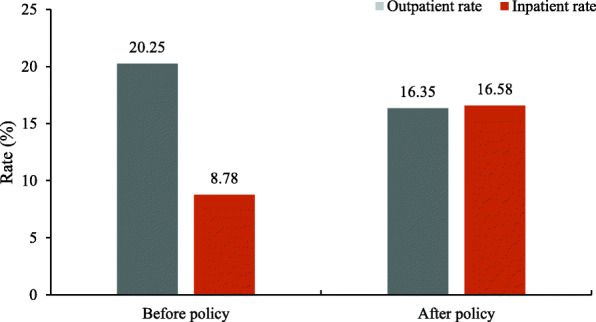
Changes of health services utilization before and after policy.

Table 1 shows the basic characteristics of the study sample used in the statistical analysis. There were 48.87 % of participants before policy implementation and 51.13 % afterwards. The age of participants was concentrated in the group of those 51–70 years old, and the percentage was 64.80 %. The education level of participants was mainly at elementary school and below (75.87 %), while 24.13 % of participants were educated beyond middle school. Most of the participants were living with others (78.90 %), were nonsmokers (73.04 %), did not consume alcohol (67.62 %), and presented no disability (80.97 %). A total of 55.20 % of the participants had chronic diseases. Figure 1 shows that 20.25 % (95 % CL: 19.54, 20.98) of the rural participants had OHSU before the introduction of the ISMI policy; this significantly decreased to 16.35 % (95 % CL: 15.71, 17.00) afterwards (χ^2^ = 63.10, *P* < 0.001). Meanwhile, the rate of IHSU for the rural participants increased from 8.78 % (95 % CL: 8.29, 9.30) before the introduction of the social health insurance policy to 16.58 % (95 % CL: 15.93, 17.24) afterwards (χ^2^ = 338.28, *P* < 0.001).

Tables 2 and 3 present the unadjusted association between basic characteristics and health services utilization, using the entire sample. Female gender; living alone; sleeping for less than 6 h/night; and having disability, chronic pain, and/or chronic disease were strongly associated with OHSU and IHSU. Education and economic status were negatively associated with OHSU and IHSU, while age was positively associated with OHSU and IHSU.

**Table 2:** Distribution of outpatient health services utilization

Variables	Group	Outpatient rate		χ^2^	***P***
		No	Yes		
**Demographics**					
Sex	Male	9,712(84.04)	1,844(15.96)	76.51	<0.001
	Female	10,515(79.74)	2,672(20.26)		
Age (years)	45-50	3,560 (82.81)	739 (17.19)	4.17	0.244
	51-60	6,986 (81.69)	1,566 (18.31)		
	61-70	6,107 (81.45)	1,391 (18.55)		
	≥71	3,583 (81.36)	821 (18.64)		
Education	Illiterate	6,110 (80.55)	1,475 (19.45)	22.65	<0.001
	≤Elementary school	9,130 (81.55)	2,066 (18.45)		
	≥Middle school	4,988 (83.69)	972 (16.31)		
Economic status	Low	4,985 (81.22)	1,153 (18.78)	3.62	0.164
	Middle	10,009 (81.72)	2,239 (18.28)		
	High	5,039 (82.53)	1,067 (17.47)		
**Life style**					
Living status	Live with others	16,031 (81.93)	3,536 (18.07)	1.96	0.161
	Live alone	4,205 (81.08)	981 (18.92)		
Sleeping hours	7-8 hours	7,532 (84.42)	1,390 (15.58)	85.96	<0.001
	≤6 hours	9,930 (79.55)	2,553 (20.45)		
	>8 hours	2,774 (82.86)	574 (17.14)		
Smoking status	No	11,655 (80.33)	2,853 (19.67)	7.95	0.005
	Yes	4,375 (82.11)	953 (17.89)		
Alcohol consumption	No	13,428 (80.25)	3,304 (19.75)	77.48	<0.001
	Yes	6,800 (84.87)	1,212 (15.13)		
**Health status**					
Disability	No	14,263 (82.61)	3,003 (17.39)	44.50	<0.001
	Yes	3,169 (78.11)	888 (21.89)		
Body pain	No	11,077 (86.97)	1,660 (13.03)	478.40	<0.001
	Yes	9,159 (76.22)	2,857 (23.78)		
Chronic diseases	No	9,791 (88.24)	1,305 (11.76)	567.35	<0.001
	Yes	10,445 (76.48)	3,212 (23.52)

**Table 3:** Distribution of inpatient health services utilization

Variables	Group	Inpatient rate		χ^2^	***P***
		No	Yes		
**Demographics**					
Sex	Male	10,157 (87.55)	1,444 (12.45)	2.12	0.145
	Female	11,491 (86.93)	1,727 (13.07)		
Age (years)	45-50	4,005 (92.67)	317 (7.33)	431.92	<0.001
	51-60	7,736 (90.12)	848 (9.88)		
	61-70	6,400 (85.23)	1,109 (14.77)		
	≥71	3,517 (79.68)	897 (20.32)		
Education	Illiterate	6,517 (85.74)	1,084 (14.26)	59.33	<0.001
	≤Elementary school	9,745 (86.77)	1,486 (13.23)		
	≥Middle school	5,388 (90.03)	597 (9.97)		
Economic status	Low	5,021 (81.71)	1,124 (18.29)	270.04	<0.001
	Middle	10,786 (87.79)	1,500 (12.21)		
	High	5,611 (91.47)	523 (8.53)		
**Life style**					
Living status	Live with others	17,146 (87.50)	2,450 (12.50)	6.03	0.014
	Live alone	4,512 (86.22)	721 (13.78)		
Sleeping hours	7-8 hours	7,963 (89.16)	968 (10.84)	54.08	<0.001
	≤6 hours	10,716 (85.77)	1,778 (14.23)		
	>8 hours	2,979 (87.51)	425 (12.49)		
Smoking status	No	12,682 (87.19)	1,864 (12.81)	58.16	<0.001
	Yes	4,888 (91.11)	477 (8.89)		
Alcohol consumption	No	14,353 (85.53)	2,428 (14.47)	133.00	<0.001
	Yes	7,293 (90.75)	743 (9.25)		
**Health status**					
Disability	No	15,613 (90.12)	1,712 (9.88)	214.39	<0.001
	Yes	3,341 (82.01)	733 (17.99)		
Body pain	No	11,666 (91.22)	1,123 (8.78)	376.96	<0.001
	Yes	9,992 (82.99)	2,048 (17.01)		
Chronic diseases	No	10,177 (91.51)	944 (8.49)	331.67	<0.001
	Yes	11,481 (83.75)	2,227 (16.25)		

Table 4 summarizes the effect of social medical insurance policy on the OHSU and IHSU when adjusting for other confounding factors by two GLMMs. In general, the most noticeable findings were that the utilization of inpatient health services nearly doubled after the introduction of the social health insurance policy compared with beforehand (OR: 1.78, 95 % CL: 1.57, 2.03). However, the rate of OHSU decreased by 31 % (OR: 0.69, 95 % CL: 0.62, 0.77) after the introduction of the ISMI policy.

**Table 4:** Effect of integration of social medical insurance policy on health services utilization

Variables	Outpatient				Inpatient			
	OR	95% CL		***P***	OR	95% CL		***P***
		Lower	Upper			Lower	Upper	
**Main variable**								
After policy	0.69	0.62	0.77	<0.001	1.78	1.57	2.03	<0.001
**Control variables**								
Female	1.06	0.94	1.20	0.350	0.85	0.73	0.99	0.036
Age (51-60 years)	1.01	0.90	1.13	0.924	1.18	1.00	1.38	0.045
Age (51-60 years)	1.01	0.89	1.14	0.886	1.76	1.49	2.07	<0.001
Age (≥71 years)	0.97	0.84	1.13	0.712	2.39	1.98	2.88	<0.001
≤Elementary school	1.10	0.99	1.21	0.063	1.17	1.04	1.32	0.012
≥Middle school	1.10	0.96	1.25	0.162	1.09	0.92	1.29	0.305
Middle economic status	0.97	0.87	1.09	0.656	0.87	0.76	0.99	0.034
High economic status	0.92	0.80	1.05	0.228	0.93	0.79	1.09	0.374
Live alone	1.02	0.92	1.12	0.763	0.94	0.83	1.07	0.355
Sleeping ≤6 hours	1.21	1.11	1.33	<0.001	1.09	0.97	1.22	0.150
Sleeping >8 hours	1.04	0.91	1.18	0.565	1.10	0.94	1.29	0.244
No smoking	0.94	0.83	1.06	0.305	0.94	0.80	1.10	0.439
No alcohol consumption	0.79	0.72	0.88	<0.001	0.63	0.55	0.72	<0.001
Disability	1.04	0.94	1.15	0.461	1.42	1.26	1.60	<0.001
Body pain	1.95	1.79	2.13	<0.001	1.38	1.24	1.54	<0.001
Chronic diseases	1.97	1.80	2.16	<0.001	2.08	1.85	2.34	<0.001

*OR* Odds Ratios, confidence limits *CL*

Unlike the results based on the univariate analysis reported in Tables 2 and 3, there was no significant association between age, education, economic status, living status, smoking, disability, and OHSU. Participants sleeping for less than 6 h/night utilized more outpatient health services than participants sleeping 7‒8 h/night (OR: 1.21, 95 % CL: 1.11, 1.33). Similarly, participants with pain (OR: 1.95, 95 % CL: 1.79, 2.13) and/or chronic diseases (OR: 1.97, 95 % CL: 1.80, 2.16) utilized more outpatient health services. Table 4 also indicates that the rate of IHSU gradually increased with age, education, and having a disability or chronic pain and/or disease, while female gender, moderate economic status, and not drinking were shown to be protective factors against IHSU (*P* < 0.05).

### Change in inequity

**Fig. 2:**
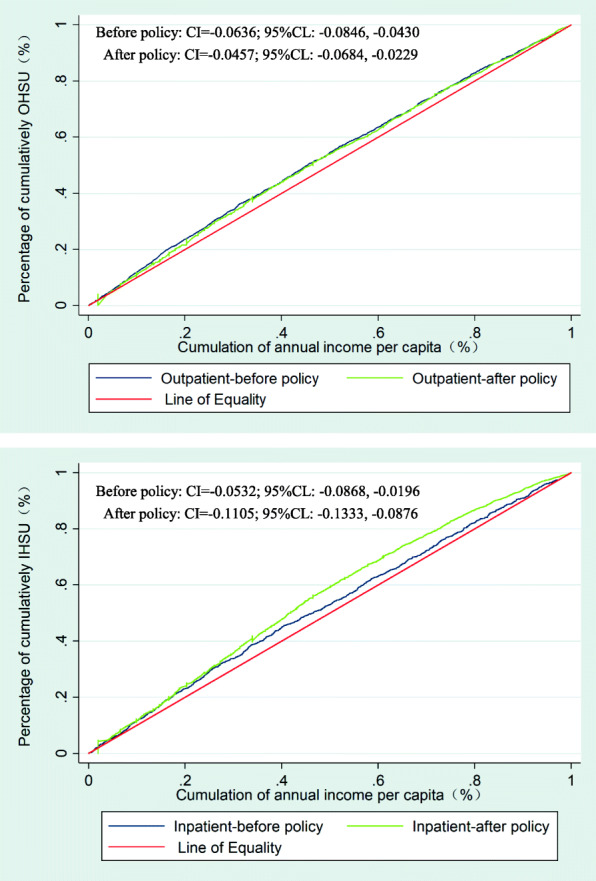
Concentration curves of outpatient and inpatient health services utilization before and after policy.

Figure 2 shows that, before the ISMI policy, concentration curves for both OHSU and IHSU lay significantly above the line of equality, indicating that OHSU and IHSU were more concentrated among the lower-income participants. After the implementation of the policy, the concentration curve of OHSU was closer to the line of equality, while the concentration curve of IHSU was farther away from the line of equality. This mean that the ISMI policy had a positive effect in terms of reducing the inequality in OHSU. In addition, the CI of OHSU decreased significantly from − 0.0636 (95 % CL: −0.0846, − 0.0430) before policy implementation to − 0.0457 (95 % CL: −0.0684, − 0.0229) afterwards; the CI of IHSU increased significantly from − 0.0532 (95 % CL: −0.0868, − 0.0196) before policy implementation to − 0.1105 (95 % CL: −0.1333, − 0.0876) afterwards. The change in the CIs indicates that the inequality of OHSU and IHSU remained in favor of lower-income participants after the implementation of the policy.

Overall, in terms of the direction of the CIs, the introduction of the ISMI policy increasingly influenced the population with low-economic status utilizing outpatient and inpatient health services. In terms of the magnitude of the CIs, the policy had a positive effect on reducing inequalities in OHSU, bringing OHSU closer to the equity line; however, it is worth noting that the policy increased inequalities in the IHSU, moving the utilization of inpatient health services further away from the equity line.

 By decomposing the concentration indexes of the OHSU and IHSU, socioeconomic inequalities were decomposed into the relative contributions of each determinant. The detailed information on elasticity, contributions of each determinant to CI, and pure percentage contributions of determinants to the socioeconomic inequality in OHSU and IHSU are reported in the additional files 1 and 2. Positive elasticity, such as health status indicated by having chronic disease, was significantly associated with the occurrence of OHSU and IHSU, whilst negative elasticity, such as smoking status, suggested the non-smoker decreased the occurrence of OHSU and IHSU. For OHSU before and after policy, we found that having pain, economic status, and having chronic disease had the largest (before: 34.76 %; after: 39.32 %), second-largest (before: 21.82 %; after: 22.87 %) and third largest (before: 16.12 %; after: 16.17 %) contributions, respectively, to the inequality of OHSU. For IHSU before and after policy, age (before: 33.22 %; after: 25.87 %) made major contributions to the inequality of IHSU. Finally, as shown in Table 5, it was determined that the HI of occurring OHSU decreased from − 0.0284 before the implementation of the ISMI policy to − 0.0171 afterwards, indicating that the inequity of OHSU was reduced after the implementation of the ISMI policy. The HI of IHSU increased from − 0.0066 before policy implementation to − 0.0595 afterwards, indicating that more lower-economic status participants utilized inpatient services after the ISMI policy.

**Table 5:** Decomposition analysis of concentration index on respondents’ health services utilization before and after policy

Variables	Outpatient				Inpatient			
	Before policy		After policy		Before policy		After policy	
	Contribution to CI	%	Contribution to CI	%	Contribution to CI	%	Contribution to CI	%
CI	-0.0636	100	-0.0457	100	-0.0532	100	-0.1105	100
Needs variables	-0.0352	55.0997	-0.0286	62.4756	-0.0466	87.5468	-0.0510	46.0528
Economic status	-0.0138	21.8262	-0.0104	22.8630	0.0001	-0.2523	-0.0150	13.6533
Other variables	-0.0032	4.9824	0.0046	-9.8942	-0.002	3.9403	-0.0138	12.4353
Residuals	-0.0114	18.0917	-0.0113	24.5556	-0.0047	8.7652	-0.0307	27.8586
HI	-0.0284	-	-0.0171	-	-0.0066	-	-0.0595	-

*CI* Concentration Index, *%* Pure percentage contributions of determinants to the socioeconomic inequality in inpatient health services utilization; Needs variables include sex, age, disability, pain and chronic diseases, other variables include education, living, smoking and drinking status, the detailed values of Contribution to CI and pure percentage for each variable are shown in the additional files 1 and 2, *HI* Horizontal inequity index.

## Discussion

Despite China conducting a series of health reforms over the past two decades and substantial improvements to health system performance [33–35], few studies have examined the impact of the inequity of ISMI on health services utilization, especially among vulnerable groups. This study was conducted in order to analyze the specific magnitude of changes in the equity of outpatient and inpatient health services utilization among the middle-aged and elderly in rural China before and after the introduction of the ISMI policy. In this study, we found that the rate of OHSU did not increase; it changed from 20.25 % before the introduction of the ISMI policy to 16.35 % afterwards. However, the HI of OHSU was negative and the value decreased from 0.0284 before the implementation of the policy to 0.0171 afterwards, indicating that the inequity of OHSU was reduced after the policy came into effect and that the population sub-group with low economic status had more OHSU than their high-economic-status counterparts did. This is consistent with the findings of a previous study examining the effect of medical insurance policy on OHSU, which found no significant relationship between them [36]. This confirms that the ISMI policy focuses on inpatient service rather than outpatients to achieve its principal goal of preventing catastrophic health expenditure. However, it differs from Wagstaff’s study in that medical insurance policy would increase outpatient service utilization [37]. Our study focused on the data collected from households survey in 29 provinces of China, while Wagstaff’s study focused on the data collected from program administrators, health facilities, and households survey in 17 provinces of China. The differences in data collection, study sample, and population may have contributed to the different findings, and, thus, further research is needed to verify this finding.

This study also proved that the rate of IHSU increased: it changed from 8.78 % before the introduction of the ISMI policy to 16.58 % afterwards. This is consistent with most studies on medical insurance policy increasing the utilization of inpatient health services [38–41]. The HI of IHSU was negative and increased from 0.0066 before the implementation of the policy to 0.0595 afterwards, indicating that the ISMI increased the rate of IHSU and that more rural, lower- economic participants utilized inpatient care after it was implemented. This is consistent with other studies that reported that the expansion of health insurance coverage increases access to health services utilization among the poor [42, 43]. The purpose of the ongoing reform of SMI is to help the rural, low- economic population to overcome the disease‒poverty trap by providing a certain level of financial protection and medical assistance.

Health equity and the social determinants of health are at the forefront of contemporary health-related research. As health inequities are avoidable products of social injustice, improving health equity is a major goal of many national governments and international organizations [44]. Measuring health equity is a key step in promoting the opportunity for all people, regardless of their social background or status, to live long and healthy lives, and in achieving the goal of a healthy China [20]. Currently, the integration reform is still in its infancy and faces many challenges due to the lack of national guidance. Therefore, conducting research on the effects of integration policy implementation is timely and provides policy-makers with an important resource. In total, all of CI values and HI values we calculated were negative, which means that the lower- economic participants had more utilization than higher- economic participants in rural areas of China; thus, the effect of the ISMI policy is in alignment with the original intention.

Some limitations of our study must be acknowledged. First, because we focused on individuals aged 45 or older due to data limitations, future studies should expand the analysis to include younger individuals. In addition, the availability of measured determinants of health services utilization were limited by the pre-specified questions in the survey, and there could be some potential unobserved confounding factors for which we did not control, such as the level of the medical services or the distance of the participants’ home from the medical services. Finally, using cross-sectional data does not allow for any causal conclusions to be drawn. Despite these limitations, we believe that the current study provides new insights for understanding the benefit of social medical insurance for health services utilization in rural China. We also expect the Chinese experience of reforming social insurance policy for rural Hukou residents to provide valuable lessons for developing countries that are also looking to establish or reform their own social insurance schemes.

## Conclusions

In summary, the ISMI policy had a positive effect on improving the rate of IHSU but not on the rate of OHSU. This is in line with the ISMI policy’s original intention to focus on inpatient services rather than outpatients to achieve its principal goal of preventing catastrophic health expenditure. Moreover, the ISMI policy had a positive effect on reducing the inequity of OHSU but a negative effect on decreasing the inequity of IHSU; thus, further research is needed to verify this change. This research on the effects of integration policy implementation may be useful to policy makers and has important policy implications for other developing countries facing similar challenges on the road to universal health coverage.

## Supplementary information

Additional file 1 Decomposition of concentration index of health services utilization before policy.

Additional file 2 Decomposition of concentration index of health services utilization after policy.

## Data Availability

The datasets generated and analyzed during the current study are available at http://charls.pku.edu.cn/.

## Abbreviations

CHARLS: China Health and Retirement Longitudinal Study
CI: Concentration Index
CL: Confidence Limits
HI: Horizontal inequity index
IHSU: Inpatient Health Service Utilization
ISMI: Integration of Social Medical Insurance
NCMS: New Rural Cooperative Medical Scheme
OHSU: Outpatient Health Service Utilization
OR: Odds Ratio
SMI: Social Medical Insurance
URBMI: Urban Residents’ Basic Medical Insurance
