# Supplementary material for: The benefits of an integrated social medical insurance for health services utilization in rural China: evidence from the China health and retirement longitudinal study
Source: Int J Equity Health. 2021 May 24;20:126. doi: 10.1186/s12939-021-01457-8 (PMC8145815; doi:10.1186/s12939-021-01457-8)
Supplement: Supplementary file 1 — Decomposition of concentration index of health services utilization before policy. [file 12939_2021_1457_MOESM1_ESM.docx]

Additional file 1 Decomposition of concentration index of health services utilization before policy

| **Variables** | **Outpatient** | | |  | **Inpatient** | | |
| --- | --- | --- | --- | --- | --- | --- | --- |
|  | **Elasticity** | **Contribution to CI** | **%** |  | **Elasticity** | **Contribution to CI** | **%** |
| **Demographics** |  |  |  |  |  |  |  |
| Female | 0.0259 | 0.0000 | -0.07 |  | -0.2070 | -0.0003 | 0.65 |
| Age (years) |  |  |  |  |  |  |  |
| 51-60 | -0.0068 | -0.0003 | 0.40 |  | 0.0048 | 0.0002 | -0.34 |
| 61-70 | 0.0163 | -0.0014 | 2.16 |  | 0.1025 | -0.0086 | 16.11 |
| ≥71 | 0.0024 | -0.0004 | 0.55 |  | 0.0640 | -0.0093 | 17.45 |
| ≤Elementary school | 0.0180 | 0.0002 | -0.27 |  | 0.0416 | 0.0004 | -0.69 |
| ≥Middle school | -0.0022 | -0.0003 | 0.45 |  | 0.0029 | 0.0004 | -0.71 |
| Economic status | | |  |  |  |  |  |
| Medium | -0.0286 | 0.0094 | -14.73 |  | -0.0223 | 0.0073 | -13.75 |
| High | -0.0391 | -0.0232 | 36.55 |  | -0.0121 | -0.0072 | 13.50 |
| **Life style** |  |  |  |  |  |  |  |
| Living alone | -0.0066 | 0.0000 | -0.03 |  | -0.1908 | 0.0006 | -1.11 |
| Sleeping hours |  |  |  |  |  |  |  |
| ≤6 hours | 0.0693 | -0.0022 | 3.38 |  | 0.0369 | -0.0011 | 2.12 |
| >8 hours | 0.0125 | -0.0003 | 0.54 |  | 0.0192 | -0.0005 | 1.00 |
| No smoking | -0.0211 | 0.0005 | -0.80 |  | -0.0131 | 0.0003 | -0.59 |
| No alcohol consumption | -0.0623 | -0.0011 | 1.71 |  | -0.1158 | -0.0021 | 3.91 |
| **Health status** |  |  |  |  |  |  |  |
| Having disability | 0.0053 | -0.0007 | 1.17 |  | 0.0439 | -0.0063 | 11.76 |
| Pain | 0.1918 | -0.0221 | 34.76 |  | 0.0954 | -0.0111 | 20.79 |
| Having chronic disease | 0.3596 | -0.0103 | 16.12 |  | 0.3907 | -0.0112 | 21.12 |

Abbreviations: *CI* Concentration Index; *%* Pure percentage contributions of determinants to the socioeconomic inequality in outpatient and inpatient health services utilization.
