# Supplementary material for: The benefits of an integrated social medical insurance for health services utilization in rural China: evidence from the China health and retirement longitudinal study
Source: Int J Equity Health. 2021 May 24;20:126. doi: 10.1186/s12939-021-01457-8 (PMC8145815; doi:10.1186/s12939-021-01457-8)
Supplement: Supplementary file 2 — Decomposition of concentration index of health services utilization after policy. [file 12939_2021_1457_MOESM2_ESM.docx]

Additional file 2 Decomposition of concentration index of health services utilization after policy

| **Variables** | **Outpatient** | | |  | **Inpatient** | | |
| --- | --- | --- | --- | --- | --- | --- | --- |
|  | **Elasticity** | **Contribution to CI** | **%** |  | **Elasticity** | **Contribution to CI** | **%** |
| **Demographics** |  |  |  |  |  |  |  |
| Female | 0.1597 | -0.0052 | 11.32 |  | -0.2398 | 0.0078 | -7.03 |
| Age (years) |  |  |  |  |  |  |  |
| 51-60 | -0.0080 | -0.0003 | 0.58 |  | 0.0852 | 0.0028 | -2.56 |
| 61-70 | -0.0644 | 0.0006 | -1.36 |  | 0.1806 | -0.0017 | 1.55 |
| ≥71 | -0.0395 | 0.0049 | -10.65 |  | 0.2411 | -0.0297 | 26.88 |
| ≤Elementary school | 0.0295 | 0.0003 | -0.69 |  | 0.0624 | 0.0007 | -0.61 |
| ≥Middle school | 0.0307 | 0.0064 | -13.93 |  | 0.0166 | 0.0034 | -3.12 |
| Economic status | | |  |  |  |  |  |
| Medium | -0.0301 | -0.0092 | 20.23 |  | -0.0517 | -0.0158 | 14.34 |
| High | -0.0013 | -0.0012 | 2.64 |  | 0.0008 | 0.0008 | -0.68 |
| **Life style** |  |  |  |  |  |  |  |
| Living alone | 0.0619 | -0.0004 | 0.92 |  | 0.0964 | -0.0007 | 0.59 |
| Sleeping hours |  |  |  |  |  |  |  |
| ≤6 hours | 0.0841 | -0.0026 | 5.76 |  | 0.0319 | -0.0010 | 0.90 |
| >8 hours | -0.0142 | 0.0012 | -2.67 |  | 0.0139 | -0.0012 | 1.08 |
| No smoking | -0.0038 | -0.0007 | 1.60 |  | -0.0101 | -0.0019 | 1.75 |
| No alcohol consumption | 0.0029 | 0.0004 | -0.89 |  | -0.0932 | -0.0131 | 11.83 |
| **Health status** |  |  |  |  |  |  |  |
| Having disability | 0.0197 | -0.0032 | 7.10 |  | 0.0734 | -0.0121 | 10.91 |
| Pain | 0.2842 | -0.0180 | 39.32 |  | 0.1481 | -0.0094 | 8.47 |
| Having chronic disease | 0.1884 | -0.0074 | 16.17 |  | 0.2209 | -0.0087 | 7.84 |

Abbreviations: *CI* Concentration Index; *%* Pure percentage contributions of determinants to the socioeconomic inequality in outpatient and inpatient health services utilization.
